# Supplementary material for: Relation between motor competence and academic achievement: The mediating role of fitness and cognition in boys and girls
Source: PLoS One. 2025 Nov 7;20(11):e0314948. doi: 10.1371/journal.pone.0314948 (PMC12594380; doi:10.1371/journal.pone.0314948)
Supplement: Attachment — Submitted filename: Response to Reviewers.docx [file pone.0314948.s002.docx]

Professor Helmi Chaabène

Academic Editor, PLOS ONE

Dear Academic Editor,

Please find enclosed a revised version of our manuscript “Relation between motor competence and academic achievement: the mediating role of fitness and cognition in boys and girls” (PONE-D-24-52528). We would like to thank you for giving us the opportunity to revise and improve our manuscript again; we also thank the reviewers for the thoughtful and constructive comments. We have considered the suggestions and have incorporated them into the revised manuscript, and as a result, we believe our manuscript is stronger. An itemized point-by-point response to the reviewers’ comments is shown below. The locations of the modifications made in the manuscript are detailed (page number, line number), as indicated in the file with track changes.

Thank you for your attention to this matter.

Abel Ruiz-Hermosa (corresponding author).

Social and Health Research Center. University of Castilla-La Mancha, Cuenca, Spain.

**Journal Requirements:**

When submitting your revision, we need you to address these additional requirements.

1.Please ensure that your manuscript meets PLOS ONE's style requirements, including those for file naming. The PLOS ONE style templates can be found at:

<https://journals.plos.org/plosone/s/file?id=wjVg/PLOSOne_formatting_sample_main_body.pdf>

<https://journals.plos.org/plosone/s/file?id=ba62/PLOSOne_formatting_sample_title_authors_affiliations.pdf>

**Authors' response:** Thank you very much for your feedback. We have reviewed the manuscript, and its format has been adjusted in accordance with the style templates provided.

2. In the ethics statement in the Methods, you have specified that verbal consent was obtained. Please provide additional details regarding how this consent was documented and witnessed, and state whether this was approved by the IRB.

**Authors' response**: Thank you very much for your comment. We have revised the relevant paragraph to provide a more detailed explanation of how verbal consent was obtained. The revised paragraph now reads as follows (page 8, line 187):

*The study protocol was approved by the Clinical Research Ethics Committee of the ‘Virgen de la Luz’ Hospital in Cuenca (REG: 2016/PI021). Before data collection, an informational session was conducted in each classroom to explain the study’s objectives, the voluntary nature of participation, the anonymity of responses, and the right to withdraw at any time without consequences. These aspects were communicated in age-appropriate language to ensure the children’s understanding. Following this explanation, each child was explicitly asked to give their verbal consent, in accordance with the procedure approved by the ethics committee. This consent was given in the presence of the classroom teacher, the responsible researchers, and the children’s peers, who served as witnesses. In addition, written informed consent was obtained from the parents or legal guardians of all participants.*

3. We note that the grant information you provided in the ‘Funding Information’ and ‘Financial Disclosure’ sections do not match.

When you resubmit, please ensure that you provide the correct grant numbers for the awards you received for your study in the ‘Funding Information’ section.

**Authors' response:** Thank you very much for your helpful correction. We have carefully revised the ‘Funding Information’ and ‘Financial Disclosure’ sections, and they now align accordingly.

4. Thank you for stating the following financial disclosure:

The “Instituto de Salud Carlos III (FIS PI16/01919) funded this study. Additional funding was obtained from the Research Network on Preventative Activities and Health Promotion (RD12/0005/0009). Furthermore, Andrés Redondo-Tébar and Abel Ruiz-Hermosa are postdoctoral researchers funded by the Margarita Salas Fellowship through the University of Castilla-La Mancha “Next Generation EU” (2022-POST-21124 and 2021-MS-20547, respectively).”

Please state what role the funders took in the study.  If the funders had no role, please state: "The funders had no role in study design, data collection and analysis, decision to publish, or preparation of the manuscript."

If this statement is not correct you must amend it as needed.

Please include this amended Role of Funder statement in your cover letter; we will change the online submission form on your behalf.

**Authors' response:** In ‘Financial disclosure’ section, we have stated that: "The funders had no role in study design, data collection and analysis, decision to publish, or preparation of the manuscript”. This information has also been incorporated into the cover letter.

5. In the online submission form, you indicated that “The data underlying the results presented in the study are available upon reasonable request to the corresponding author”.

All PLOS journals now require all data underlying the findings described in their manuscript to be freely available to other researchers, either 1. In a public repository, 2. Within the manuscript itself, or 3. Uploaded as supplementary information.

This policy applies to all data except where public deposition would breach compliance with the protocol approved by your research ethics board. If your data cannot be made publicly available for ethical or legal reasons (e.g., public availability would compromise patient privacy), please explain your reasons on resubmission and your exemption request will be escalated for approval.

**Authors' response:** We sincerely appreciate your comment. The minimal anonymized dataset required to replicate our study findings has been published in the ZENODO public repository and is openly accessible at the following URL: <https://zenodo.org/records/17070143> (DOI: 10.5281/zenodo.17070143).

6. PLOS requires an ORCID iD for the corresponding author in Editorial Manager on papers submitted after December 6th, 2016. Please ensure that you have an ORCID iD and that it is validated in Editorial Manager. To do this, go to ‘Update my Information’ (in the upper left-hand corner of the main menu), and click on the Fetch/Validate link next to the ORCID field. This will take you to the ORCID site and allow you to create a new iD or authenticate a pre-existing iD in Editorial Manager.

**Authors' response:** Done.

Additional Editor Comments:

Dear authors,

I'm sorry for the delay in providing our feedback. We were in the process of seeking additional reviewers after identifying a potential conflict of interest between at least one of the initial reviewers and the research team. Unfortunately, we were not very successful in recruiting new reviewers. To avoid any further delay, I have reviewed the manuscript myself. In my assessment, the study is well designed and executed. The topic is both interesting and relevant, and to the best of my knowledge, it has not been previously addressed, making it a novel contribution. The sample size is appropriate, and the main findings are compelling. They lay a strong foundation for future research to replicate the results and explore the potential role of sex as a moderating variable.

Please make sure to address all reviewers’ comments and suggestions in a point-by-point response and implement the corresponding amendments to the manuscript where appropriate.

I'm looking forward to receive the revised version.

All the best,

Helmi Chaabène

**Authors' response:** Thank you very much for your feedback.

**Response to Reviewer 1 Comments:**

Reviewer #1: I would like to thank authors for this interesting study. Academic achievement is an important subject, and it has a relation with physical fitness. I believe this study has many strengths. First, it has a very good sample size, and tests and measurements are appropriate. In addition, it has strong results and discussion sections. However, authors need to report reliability and validity of tests and measurements. In addition, this study needs a stronger conclusion section with future perspectives and practical applications. Lastly, this study needs more up to date (2022 and above literature review. I look forward to seeing edited version of this manuscript. Best regards.

**Authors' response:** Thank you very much for your feedback. To report the reliability and validity of the tests and measurements, we have added the following sentences in the 'Instruments and Study Variables' section—specifically when defining each battery or tool—to provide evidence of their reliability and validity:

First paragraph (page 8, line 211): *All measurements were conducted by trained researchers who followed standardized protocols to ensure consistency and accuracy in data collection. The evaluators were blinded to the participants' group assignments.*

Relating to Movement Assessment Battery for Children-Second (page 9, line 225): *This battery has shown strong content and construct validity for assessing motor competence in children, with test–retest reliability coefficients above 0.80 [1].*

Relating to ALPHA-Fitness test battery (page 10, line 256): *This battery has demonstrated high criterion validity and reliability across European school populations. Specifically, the 20-meter shuttle run, 4×10-meter shuttle run, handgrip strength test, and standing broad jump test have all been validated as reliable field-based assessments of cardiorespiratory fitness, speed/agility, and muscular strength, respectively [26,27].*

Relating to NIH Toolbox software (page 12, line 289): *This tool has demonstrated high internal consistency (Cronbach’s alpha > 0.80), excellent test–retest reliability, and strong convergent validity with standardized neuropsychological measures in pediatric populations [31,32].*

We have revised the Conclusions section to enhance its clarity and impact, adding content on future perspectives and practical applications. The updated Conclusions section now reads as follows (page 29, line 679):

***Conclusions***

*Our findings indicate that the association between GMC and AA in children is largely explained by improvements in CRF, S/A, and EF, with distinct mediation patterns by sex. These results emphasize the importance of implementing school-based programs that integrate motor skill development with both fitness and cognitive training. Specifically, boys may benefit more from cognitively demanding, perceptual–motor activities, while girls may respond better to interventions prioritizing physical fitness enhancement.*

*From a practical perspective, educators and policymakers should prioritize daily opportunities for structured physical activity that go beyond regular physical education classes, incorporating aerobic, coordinative, and cognitively engaging tasks. Such approaches could foster academic performance, reduce educational inequalities, and contribute to healthier developmental trajectories.*

*Future research should examine the long-term effects of integrated motor–cognitive interventions on AA, explore the neurobiological mechanisms underlying these associations, and consider additional mediators such as sleep, attention regulation, and motivation. By framing motor competence not only as a physical education outcome but as a multidisciplinary strategy, schools can promote cognitive development, academic success, and lifelong health.*

Finally, numerous references in the resubmitted manuscript have been reviewed and updated to reflect more current and relevant sources. The new references have been highlighted in red, both in citation [6] and in the references list at the end of the manuscript.

**Response to Reviewer 2 Comments:**

Reviewer #2: Este artículo entrega respuestas a una serie de inquietudes que se presentan en el campo de la educación. La indagación tiene un correcto desarrollo que ayuda a ampliar el conocimiento respecto a la temática en cuestión. Por tanto, el presente artículo cumple correctamente las etapas en cuanto a forma y fondo.

**Authors’ response:** We sincerely thank you for your review and the feedback.

**Response to Reviewer 3 Comments:**

Reviewer #3: REVIEW REPORT (PONE-D-24-52528)
Relation between motor competence and academic achievement: the mediating role of fitness and cognition in boys and girls.

**ABSTRACT**

- Please adjust the acronym “AA” that was used without definition and review the presentation of the percentages and effect coefficients for adequate contextualization, focusing more on the main findings in interpretative language (for example, “cognitive mediation was more important in boys, while fitness was more expressive in girls”).

**Authors' response:** Thank you very much for your valuable comment. We have replaced the acronym “AA” with its full term throughout the abstract (page 2, line 67 and page 3, line 71). Additionally, we have revised the presentation of percentages and effect sizes to ensure clearer contextualization, placing greater emphasis on the main findings using more interpretative language. The revised abstract now reads as follows (page 2, line 49):

***Abstract***

***Introduction.*** *Gross motor competence is positively associated with academic achievement in schoolchildren, potentially mediated by fitness and cognition. However, the extent to which these mediators contribute—and whether effects differ by sex—remains unclear. This study explored the mediating roles of specific fitness components and executive function in the relationship between gross motor competence and academic achievement, considering sex differences.*

***Methods.*** *This cross-sectional study included 562 Spanish schoolchildren aged 9-11 years (293 girls). Gross motor competence was evaluated using the Movement Assessment Battery for Children-Second Edition; fitness components (cardiorespiratory fitness, speed/agility, upper and lower body strength) through the ALPHA-Fitness test battery; executive function using the NIH Toolbox Battery; and academic achievement from school grades in language and mathematics. Serial multiple mediation models were applied using the PROCESS macro in SPSS, adjusted for age, BMI, and maternal education level. Analyses were conducted for the total sample and by sex.*

***Results.*** *Both fitness and executive function partially mediated the relationship between gross motor competence and academic achievement. In the total sample, direct effects explained most of the association (51-73%), followed by the cognitive pathway (20-31%), fitness pathway (11-19%), and multiple pathway—gross motor competence, fitness, executive function, and academic achievement—(4-9%). Sex-specific analyses showed that cognitive mediation was predominant in boys, accounting for over half of the total effect (56–69%), with no direct effect observed. In contrast, fitness mediation was more relevant in girls, especially through cardiorespiratory fitness and speed/agility, each contributing up to 20% of the effect. The multiple pathway was also significant in girls.*

***Conclusions.*** *Enhancing motor competence may improve academic outcomes, partly through gains in fitness and executive function. These findings support implementing integrated school programs, tailored to sex-specific needs—emphasizing cognitively engaging activities for boys and fitness-focused strategies for girls. The cross-sectional design implies association, not causality.*

**INTRODUCTION**

- Overall, it is ok. But I believe that the hypothesis needs to be more evident, such as “it is expected that executive functions and fitness components partially mediate the relationship between motor competence and academic performance, with variations according to sex”.

**Authors' response:** Thank you for this valuable suggestion. We have incorporated a sentence stating the hypothesis at the end of the introduction, thereby strengthening the logical progression of our study and providing a natural transition to the Methods section. The final paragraph of the introduction now reads as follows (page 7, line 163):

*It is therefore necessary to conduct further in-depth research into the relationships between MC, fitness components, cognitive abilities, and AA, with a particular focus on potential sex differences. The objective of this study was to examine whether fitness and EF act as mediators in the relationship between GMC and AA, and to identify potential differences between boys and girls. Based on the reviewed literature, it is hypothesized that EF and specific fitness components partially mediate the relationship between GMC and AA, and that the magnitude of these mediations differs between boys and girls. Given the cross-sectional design, the study focuses on identifying associations rather than establishing causal relationships.*

- How does the present study differ from previous studies reporting the absence or presence of mediation? Methodological inconsistencies? Different samples or measures of physical fitness?

**Authors' response:** We appreciate the reviewer’s insightful comment. In response, we have revised the introduction to explicitly state how our study differs from previous research reporting the presence or absence of mediation. Specifically, earlier studies have shown methodological inconsistencies, including differences in the operationalization of physical fitness (e.g., relying solely on cardiorespiratory fitness or speed/agility tests), the use of heterogeneous or non-standardized measures of motor competence, and varied analytical approaches. Moreover, many studies were conducted in populations with different age ranges, cultural contexts, or did not examine mediation effects by sex. In contrast, our study simultaneously evaluates multiple physical fitness components and executive functions as mediators, applies a standardized battery of validated assessments, and examines these relationships in a large, representative sample of Spanish schoolchildren aged 9–11 years, with specific analysis by sex. These clarifications have been incorporated into the penultimate paragraph of the introduction, that now reads as follows (page 6, line 150):

*Finally, research indicates that boys and girls exhibit distinct trajectories in physical and cognitive development [19], as well as in brain activation during cognitive tasks [20]. Such differences could affect the relationship between MC and its connection to fitness, cognitive functions, and AA [21]. However, no studies have specifically analyzed the mediating role of fitness and EF by sex. Despite increasing evidence on the links between MC, fitness, EF, and AA, previous studies have relied on partial assessments, single-component fitness measures, and inconsistent analytical approaches. Critically, no research has examined multiple fitness components and EF simultaneously or explored sex-specific mediation pathways. This study addresses these gaps by providing a comprehensive analysis of how gross motor competence (GMC) relates to AA in boys and girls, using validated tests in a representative sample of Spanish schoolchildren aged 9–11 years.*

**METHODS**

- Were only students with complete data included? Were students with disabilities excluded? Please clarify.

**Authors' response:** Thank you for your questions. To clarify this point, we have inserted the following paragraph into the Methods section (page 8, line 198):

*For the analysis, only students with complete data—those who completed all assessment tests—were included. Students with disabilities were not excluded solely on the basis of disability; they were eligible if they met the inclusion criteria described in the original trial. Children were excluded if they exhibited difficulties in learning Spanish; if teachers or parents reported serious physical or mental disorders that could hinder participation in program activities; or if pediatricians identified chronic conditions—such as heart disease, diabetes, or asthma—that could prevent participation.*

- I recommend a better explanation of the z-score procedure for GMC and EF. Is it a standardization within the sample? Between age groups?

**Authors' response:** Thank you for your comments. To clarify, z-scores were computed separately for each test, in order to allow comparability across measures with different units. For each test, raw scores were standardized within age- and sex-specific groups by subtracting the group mean from each individual value and dividing by the corresponding group standard deviation. This procedure ensures that each score reflects the individual’s relative performance compared to peers of the same age and sex. The GMC Total Score was then derived as the sum of all standardized test scores. The same procedure was applied to each executive function test (inhibitory control, cognitive flexibility, and working memory) to obtain the EF Total Score. The following information have been added in “Methods” section to clarify this point:

(page 10, line 243)

*To enable comparison between tests with different measurement units, raw scores for each test (aiming-catching, static balance, and dynamic balance) were standardized into z-scores. Z-scores were calculated within age- and sex-specific groups by subtracting the group mean from the individual score and dividing by the group standard deviation. The GMC Total Score was obtained as the sum of all standardized test scores.*

(page 13, line 326)

*An EF Total Score, defined as the sum of the z-scores for inhibitory control, cognitive flexibility, and working memory, was calculated following the same standardization procedure described previously for GMC.*

- Was there standardization of grades among schools, that is, were the evaluation criteria uniform? Please include this information.

**Authors' response:** Thank you for your comment. Yes, in Spain, the curriculum, assessments, and grading criteria are standardized across all schools within the same region. No variables were controlled, as both the curriculum and the content are identical for all schools and students in that region. To clarify this point, the following information have been added in “Methods” section (page 14, line 337):

*Since schools within the same region follow a standardized curriculum, assessment system, and grading criteria, these grades are comparable across students and schools. For the analyses, the arithmetic mean of language and mathematics grades was calculated, representing each student’s academic performance over a complete school year.*

**RESULTS**

- I really believe that tables 1 and 2 should contain clearer notes about the units and meaning of the tests, such as "the higher, the better", or "shorter time = better agility". In addition, use the magnitude of the effects explicitly if the effect is small, medium or large as suggested by Cohen, especially in indirect effects.

**Authors' response:** Thanks for your corrections. In Table 1, all tests have their respective units noted in the footnotes. To clarify their meaning, the following sentence was added to the footnotes:

(page 17, line 417)

*Higher scores indicate better performance, except in Speed/Agility*, where lower scores (shorter times) indicate better performance.*

In table 2, the following information has been added to the footnotes:

(page 19, line 456)

*Higher coefficients indicate stronger correlations, except for Speed/Agility^T^, where lower coefficients reflect stronger performance (inverse variable).*

(page 19, line 458)

*Interpretation of correlation coefficients based on Cohen (1988):* *0.10-0.29, small; 0.30-0.49, medium; >0.50, large.*

In table 3, the following information has been added to the footnotes:

(page 22, line 520)

*Interpretation of effect sizes based on Cheung (2009): 0.14-0.35, small; 0.36-0.50, medium; >0.51, large.*

- The results are ok, with no evidence of artificial homogeneity or variance suppression, but ambiguity about causality is needed and to avoid undue temporal inference in a cross-sectional study. Please review this throughout the text.

**Author’s response:** We appreciate the reviewer’s observation. We have carefully reviewed the manuscript to ensure that the cross-sectional nature of our study is explicitly acknowledged throughout, avoiding any undue causal or temporal inferences. Accordingly, we have modified the wording in the Abstract, Introduction, Discussion, and Conclusions sections to use terms such as “association” or “relationship” instead of causal language (e.g., “effect”, “impact”), and we have reiterated the limitation regarding causality where appropriate.

**DISCUSSION**
- It is well written, but, in general, the authors should condense the paragraphs, avoiding repeating previous numerical results.
- The authors should moderate the first paragraph to use more accessible language instead of excessively technical. The text should convey the main message to all readers and not just the specialized ones. For example, boys showed exclusive cognitive mediation, while girls had multiple mediation pathways.
- The authors mention the general results and highlight the importance of the cognitive pathway in boys, but it is not clear why the physical components UBS and LBS did not act as mediators for them, contrary to what occurred in part with girls. Please review this, albeit objectively.
- How can PE teachers or policy makers use this evidence? This is not clear.
- The authors also inform the limitation of not measuring physical activity or sleep. Objectively, I suggest including objective information with suggestions on how to overcome this in future studies.

**Author’s response:** We sincerely appreciate your insightful comments, which have been extremely helpful in guiding the improvement of our manuscript. In response, we have thoroughly revised the Discussion section, carefully following all of your suggestions. The updated version of the Discussion is presented below (page 23, line 522):

***Discussion***

*This study is, to our knowledge, the first to apply multiple mediation models to explore how physical fitness and EF influence the link between GMC and AA in children. Our findings show that this relationship is partially explained through three distinct pathways: a fitness pathway (especially via CRF and S/A), a cognitive pathway (via EF), and a multiple pathway involving both fitness and cognition. These pathways remained significant even after adjusting for age, BMI, and maternal education level. Importantly, we observed sex-specific patterns: boys showed exclusive mediation through cognition, while girls showed several mediation pathways, including fitness and cognitive components.*

*These results align with prior studies. For example, Schmidt et al. [10] found that EF fully mediated the GMC–AA link in children using structural equation modeling, and our earlier work also highlighted the partial mediating role of EF [12]. Cadoret et al. [5] similarly observed cognitive mediation between motor proficiency and academic performance. However, only one previous study [14] specifically tested CRF as a mediator, finding no mediation—contrary to our results. This discrepancy could be explained by the different instruments and predictive methodologies used to evaluate CRF, MC, and AA, as well as changes in educational level and the transformations that occur during puberty.*

*Our results suggest that improving GMC may enhance physical fitness and cognitive abilities, ultimately benefiting academic outcomes. This was especially evident in girls, where CRF, S/A, and LBS contributed to the mediation process. These findings underscore the importance of designing motor interventions that enhance both fitness (particularly CRF and S/A) and EF, especially in educational settings targeting children aged 9 to 11.*

*Our study revealed notable sex differences in the relationship between GMC and AA. Among girls, this relationship appeared more complex, involving both direct associations and multiple mediating pathways through fitness (CRF and S/A), cognition (UBS), and combined effects (CRF, S/A, and LBS). In contrast, for boys, the relationship was mediated only through cognition, aligning with findings by Fernández-Sánchez et al. [12], but differing from Lopes et al. [11], who reported no significant sex differences. The presence of both direct and fitness-mediated associations in girls suggests a more integrated development of physical and cognitive domains compared to boys. These disparities may be explained by biological and sociocultural factors influencing motor and cognitive development [39], such as sex-specific patterns of biological maturation [40], brain development [41], and gender-related activity preferences [42]. Another possible explanation is that boys and girls may engage distinct neural circuits and molecular mechanisms when addressing the same cognitive challenges, leading to differences in strategy despite comparable overall ability [43]. Given the limited evidence focused specifically on sex differences, further research is needed.*

*When comparing the strength of the mediation pathways, we found that in the total sample, the cognitive pathway was the most influential, followed by the fitness pathway and the multiple pathway. For boys, cognition was the sole mediator, supporting the "cognitive stimulation hypothesis" [44,45]. In girls, however, the fitness pathway—particularly through CRF and S/A—was the dominant route, aligning more closely with the "cardiovascular fitness hypothesis" [46]. These differences may reflect underlying neurodevelopmental trajectories, particularly around age 9–10, when sex differences in brain maturation are especially pronounced [47].*

*Finally, our findings underscore the role of CRF and S/A as key mediators of cognitive benefits, whereas the contribution of muscular strength remains less clear. This distinction may reflect different biological and neurological mechanisms. CRF has been linked to angiogenesis in the motor cortex and increased cerebral blood flow, improving brain vascularization and supporting cognitive performance [48]. Similarly, S/A may enhance attention [49] and accelerate neural impulse conduction, thereby increasing processing speed [16]. These adaptations, together with enhanced spinal cord function, synaptogenesis, greater BDNF release, and reorganization of motor cortex representations [50], likely contribute to improved cognition [51] and, ultimately, to better AA [16]. However, the role of muscular strength, particularly UBS and LBS, remains uncertain. Evidence regarding its association with cognition and AA is mixed: while some studies report positive correlations [51,52], others find no significant relationship [53,54]. Thus, further research is required to clarify the contribution of muscular strength, and more broadly of physical fitness components, to cognitive and academic outcomes.*

***Limitations***

*This study has several limitations. First, its cross-sectional design limits causal inference. Longitudinal studies are needed to confirm the temporal direction of these relationships. Nevertheless, the temporality proposed in our models is consistent with the current knowledge about the relationship between GMC, fitness, EF, and AA [5,10,12,55,56]. Second, while school-based academic assessments are useful, they may not reflect the full range of cognitive abilities or academic skills. Third, we did not account for behavior-related factors such as physical activity or sleep, both of which can influence fitness, cognition, and academic outcomes. Future studies should objectively measure physical activity (e.g., using accelerometers) and sleep quality (e.g., actigraphy or parent-reported sleep diaries) to better understand their potential confounding or mediating roles. Finally, variables such as visual acuity or adiposity were not considered, though both may affect motor test performance [57–59].*

***Conclusions***

*Our findings indicate that the association between GMC and AA in children is largely explained by improvements in CRF, S/A, and EF, with distinct mediation patterns by sex. These results emphasize the importance of implementing school-based programs that integrate motor skill development with both fitness and cognitive training. Specifically, boys may benefit more from cognitively demanding, perceptual–motor activities, while girls may respond better to interventions prioritizing physical fitness enhancement.*

*From a practical perspective, educators and policymakers should prioritize daily opportunities for structured physical activity that go beyond regular physical education classes, incorporating aerobic, coordinative, and cognitively engaging tasks. Such approaches could foster academic performance, reduce educational inequalities, and contribute to healthier developmental trajectories.*

*Future research should examine the long-term effects of integrated motor–cognitive interventions on AA, explore the neurobiological mechanisms underlying these associations, and consider additional mediators such as sleep, attention regulation, and motivation. By framing MC not only as a physical education outcome but as a multidisciplinary strategy, schools can promote cognitive development, academic success, and lifelong health.*

**Response to Reviewer 4 Comments:**

Reviewer #4: I appreciate the opportunity to review this manuscript titled “Relation between motor competence and academic achievement: The mediating role of fitness and cognition in boys and girls”. In this study, the authors assess the mediating role of fitness and executive functions on the motor competence and academic achievement relationship. I think that a major revision is necessary before accepting it for publication.

**Author’s response:** We sincerely appreciate your thoughtful review, which has greatly contributed to improving our manuscript.

**ABSTRACT**

Although, in general, I consider that the abstract is well synthesized and provides the necessary information, it would be advisable to specify and/or define what is meant by multiple path in the results section. It is explained in the full text of the article, but if you have not read it beforehand, this aspect is not clear in the abstract.

**Authors' response:** Thank you for this valuable comment. We have added a sentence to the abstract clarifying our understanding of the term “multiple pathway” (page 3, line 71):

*In the total sample, direct effects explained most of the association (51–73%), followed by the cognitive pathway (20–31%), fitness pathway (11–19%), and multiple pathway —gross motor competence, fitness, executive function, and academic achievement— (4–9%).*

**INTRODUCTION**

The introduction is well-written, and the objectives are clearly stated. In this respect, no suggestions for improvement are made.

**Authors' response:** Thank you very much.

**METHODS**

The methodology used in the research is well explained and appropriate to achieve the objectives of the work.

**Authors' response:** Thank you very much.

**RESULTS**

- Referring to Table 1, it would be appropriate to add the effect size (Cohen's d) of the t-tests.
- The authors show in Table 2 the total and group-wise correlations for the main study variables. In relation to these results, it would perhaps be interesting to also add a statistical test (e.g. Fisher's Z-transformation) that allows to know concretely which specific correlations are statistically significantly different according to gender.

**Authors' response:** Thank you very much for your comments. In Table 1, effect sizes (Cohen’s d) have been added in the final column (page 16, line 409), and the following information has been included in the statistical analysis section and in table 1 footnotes, respectively:

(page 15, line 370)

*Effect sizes were estimated using Cohen’s d, with values below 0.20 considered negligible, 0.21-0.49 small, 0.50-0.79 medium, and ≥0.80 large [36].*

(page 20, line 416)

*Cohen’s d effect sizes (Cohen, 1988): < 0.20 no effect; 0.21-0.49, small effect; 0.50-0.79 medium effect.*

The Fisher Z-transformation test was performed, and a new symbol has been added in Table 2 (^) to indicate statistically significant differences by sex. The following information has been included in the statistical analysis section and in table 2 footnotes, respectively:

(page 15, line 376)

*A Fisher’s Z-transformation test was performed to assess differences by sex, which were statistically significant [37].*

(page 19, line 461)

*Fisher Z-Transformation* indicate statistically significant differences by sex*: ^p < 0.05; ^^p < 0.01.*

**DISCUSSION**

- The discussion is well-written. In this respect, no suggestions for improvement are made.

**Authors' response:** Thank you very much.
